# TCR Dependent Metabolic Programming Regulates Autocrine IL-4 Production Resulting in Self-Tuning of the CD8^+^ T Cell Activation Setpoint

**DOI:** 10.3389/fimmu.2020.00540

**Published:** 2020-03-31

**Authors:** Kali F. Crofts, Beth C. Holbrook, David R. Soto-Pantoja, David A. Ornelles, Martha A. Alexander-Miller

**Affiliations:** ^1^Department of Microbiology and Immunology, Wake Forest School of Medicine, Winston-Salem, NC, United States; ^2^Department of Cancer Biology, Comprehensive Cancer Center, Wake Forest School of Medicine, Winston-Salem, NC, United States; ^3^Department of Surgery, Wake Forest School of Medicine, Winston-Salem, NC, United States

**Keywords:** IL-4, metabolism, functional avidity, CD8^+^ T cell, NFAT, activation

## Abstract

The ability of T cells to sense and respond to environmental cues by altering their functional capabilities is critical for a safe and optimally protective immune response. One of the important properties that contributes to this goal is the activation set-point of the T cell. Here we report a new pathway through which TCR transgenic OT-I CD8^+^ T cells can self-tune their activation threshold. We find that in the presence of a strong TCR engagement event there is a shift in the metabolic programming of the cell where both glycolysis and oxidative phosphorylation are significantly increased. This diverges from the switch to a predominantly glycolytic profile that would be predicted following naïve T cell activation. Our data suggest this altered metabolic program results in the production of autocrine IL-4. Both metabolic pathways are required for this cytokine to be made. IL-4 signaling in the activated OT-I CD8^+^ T cell results in modulation of the sensitivity of the cell, establishing a higher activation setpoint that is maintained over time. Together these data demonstrate a novel mechanism for the regulation of IL-4 production in CD8^+^ T cells. Further, they reveal a new pathway for the self-tuning of peptide sensitivity. Finally, these studies uncover an unexpected role for oxidative phosphorylation in regulating differentiation in these cells.

## Introduction

CD8^+^ T cells are a critical component of the adaptive immune system's ability to clear virus and tumor cells. The effectiveness of these cells *in vivo* is determined by a number of parameters including quantity, array of cytokines produced, lytic activity, and peptide sensitivity. The ability of a naïve cell to acquire these attributes is dependent on an array of signals that drive differentiation of the cell, including changes in metabolism. TCR ligation on naïve cells elicits a signaling cascade that results in augmentation of cellular bioenergetic demands to provide metabolic intermediates that lead to production of ATP and support of T cell expansion (1). The glycolytic shift that occurs following activation plays a vital role in proliferation and acquisition of effector function (2–4).

The sensitivity of a CD8^+^ T cell to peptide/MHC, i.e., its functional avidity, is an important determinant of the ability of the cell to clear virus or tumor *in vivo* (5–12). Within a responding polyclonal *in vivo* elicited CD8^+^ T cell population specific for a given epitope, there are clones of divergent functional avidity (5, 13). While the importance of this property is well-established, the mechanisms responsible for determining the activation set-point are less so. The regulation of T cell sensitivity appears to be the result of both inherent and induced properties of the cell. For example, higher tetramer binding, designated as structural avidity, has been associated with increased sensitivity to peptide antigen (12, 14). This is often attributed to higher TCR affinity (12, 14), an inherent property, but can also be a result of higher order TCR organization within the membrane, an induced property (15).

In our previous studies we investigated the extent to which the inherent TCR affinity parameter governs peptide sensitivity and whether this was a restricting determinant of functional avidity. To address this we utilized a TCR transgenic mouse model which did not allow differences in TCR affinity to contribute to functional avidity. We found that high and low avidity CD8^+^ T cells could be generated following stimulation with low vs. high concentrations of peptide, respectively (16, 17). Subsequent studies suggested the decrease in avidity was associated with a decrease in the efficiency of TCR signal transduction (18).

A critical question from these findings was whether the disparate functional avidity observed in the population reflected an induced or inherent difference in the naïve pool. If the former, we reasoned that a single cell could give rise to both high and low avidity daughters. Our results revealed that the majority of cells were not pre-committed but rather could tune their peptide sensitivity in response to the level of peptide/MHC encountered (19). These findings support a model wherein CD8^+^ T cells have a window of plasticity during which avidity can be modulated; however, the activation set-point eventually becomes a fixed property of the cell as stimulation with a sub- or supraoptimal level of peptide results in death (20).

The pressing question that arose from these studies was the mechanism through which the level of peptide encountered dictated functional avidity. Cytokines are a well-established modulator of T cell function and there is evidence that they may play a role in regulating the quality of CD8^+^ T cells. For example, addition of IL-15 during the *in vitro* stimulation of CD8^+^ T cells can increase functional avidity (21). In contrast, IL-4 or 1L-13 have been reported to decrease CD8 expression and/or tetramer binding (22–27), which are likely to be associated with an increase in the amount of antigen required for activation.

Given the potential immunoregulatory role of cytokines and our finding that functional avidity is a modulatable property, we exploited our TCR transgenic model to test the hypothesis that autocrine cytokine production by CD8^+^ T cells serves as a mechanism for self-tuning of peptide sensitivity. Here we report the finding that activation with high peptide/MHC induces autocrine production of IL-4 that in turn results in altered peptide sensitivity that is a sustained property of the cell. To our knowledge, this is the first report of the ability of the level of TCR engagement to serve as a rheostat that regulates IL-4 production by CD8^+^ T cells. Strong TCR engagement was associated with metabolic reprogramming that regulated the production of autocrine IL-4. Our studies also revealed increased NFAT in the nucleus that was associated with the production of IL-4. These findings are, to our knowledge, the first to report metabolic programming dependent autocrine IL-4 production as a regulator of the activation setpoint in CD8^+^ T cells. These data reveal a novel sensing and self-tuning mechanism for establishing peptide sensitivity in CD8^+^ T cells.

## Materials and Methods

### Mice

C57BL/6 mice were obtained from The Jackson Laboratory (#000664). OT-I mice (28) on the Rag2^−/−^ background were purchased from Taconic (#2334-F). All experiments in this study comply with the institutional guidelines approved by the Wake Forest Animal Care and Usage Committee.

### Generation and Maintenance of *in vitro* CD8^+^ T Cell Lines

Splenocytes, isolated from 6 to 12 week old female C57BL/6 mice, were incubated in the presence of 10^−7^M or 10^−11^M OVA_257−264_ peptide (SIINFKEL) (Chi Scientific) or no peptide for 3 h at 37°C in a 5% CO_2_ humidified incubator. Stimulators were washed, irradiated (2,000 rads) and stained with CFSE (CFSE cell division tracker kit) (Biolegend) to allow their identification by subsequent flow cytometric analyses. OT-I Rag2^−/−^ splenocytes, isolated from 6 to 12 week old female mice were co-cultured with the irradiated C57BL/6 peptide pulsed splenocytes (1:16 ratio) in the presence of 10 U/ml recombinant human IL-2 (rhIL-2, carrier free, Biolegend) for 7 days at 37°C in a 5% CO_2_ humidified incubator. Cultures were maintained in 24-well plates in RPMI-1640 medium supplemented with 2 mM L-glutamine, 0.1 mM sodium pyruvate, 1X non-essential amino acids, 100 U/ml penicillin, 100 μg/ml streptomycin, 10 mM HEPES (Gibco), 0.05 mM 2-mercaptoethanol (Sigma Aldrich) and 10% fetal bovine serum (Atlanta Biologics). OT-I CD8^+^ T cells were restimulated weekly with irradiated peptide-pulsed C57BL/6 splenocytes and rhIL-2 (10 U/ml). For neutralization of IL-4, 5 μg/ml of anti-mouse IL-4 (clone 11B11) (Leaf Purified anti-mouse IL-4, Biolegend) or 5 μg/ml of Rat IgG1, k isotype control (BD Pharmingen Purified NA/LE Rat IgG1, K isotype control, BD Biosciences) was added to the cultures weekly during the culture set up. For calcineurin inhibition assays 100 ng/ml of Cyclosporine A (Sigma Aldrich) or 25 μM of INCA-6 (CAS 3519-82-2) (Millipore Sigma) were added to cultures. Ionomycin (Sigma Aldrich) was added at 500 ng/ml to cultures to induce intracellular Ca^2+^ increases in the cells. DMSO (Sigma Aldrich) was used as a vehicle control.

### Functional Avidity Assays

OT-I Rag2^−/−^ cells from cultures stimulated with 10^−7^M or 10^−11^M OVA pulsed stimulators were harvested on day 5 following weekly stimulation and plated in a 96-well round bottom plate (1 × 10^5^ cells/well). Cells were restimulated with graded concentrations of OVA_257−264_ peptide (10^−13^M−10^−6^M) in the presence of brefeldin A (1:1,000 dilution) (BD GolgiPlug protein transport inhibitor, BD Biosciences) for 5 h at 37°C in a 5% CO_2_ humidified incubator. Cells were pelleted and stained with Zombie Violet fixable viability kit (Biolegend) followed by staining with PerCP/Cyanine5.5 anti-mouse CD8α antibody (clone 53.6-7) (Biolegend). For intracellular cytokine staining, cells were fixed and permeabilized with Cytofix/Cytoperm (BD Biosciences) and subsequently stained with either APC anti-mouse IFNγ antibody (clone XMG1.2) (Biolegend) or APC Rat IgG1, k isotype control antibody (clone RTK2071) (Biolegend). Samples were acquired using the Fortessa X20 (BD Biosciences) and data analyzed using DIVA software (BD Biosciences).

### IFN-γ and IL-4 ELISA

Supernatant was harvested from OT-I cell cultures at 5, 12, 24, 48, and 72 h following stimulation with OVA_257−264_ peptide-pulsed stimulators in the presence of rhIL-2 (10 U/ml). IFNγ secretion was measured using the mouse IFN-γ ELISA MAX set (Biolegend). IL-4 secretion was measured using the mouse IL-4 ELISA MAX set (Biolegend). The concentration of each cytokine was calculated per manufacturer's instructions using the kit's standard.

### Anti-CD3ε Coated P815 Activation of OT-I Cells

Murine P815 cells were pulsed with titrated (60 μg−0.73 μg/ml) amounts of purified hamster anti-mouse CD3ε Ab (clone 145-2C11) (BD Pharmingen) for 1 h on ice. Cells were irradiated with 10030 rads and washed. Irradiated P815 cells (3 × 10^5^) were cultured with naïve OT-I splenocytes (3 × 10^5^) (1:1 ratio) with 10 U/ml of rhIL-2 in a humidified incubator at 37°C under 5% CO_2_ in 24-well plates. Supernatant was collected at 24, 48, and 72 h post stimulation.

### Isolation of CD8α^+^ OT-I T Cells by Positive Magnetic Selection

CD8α^+^ cells were positively selected from naïve OT-I splenocytes using CD8α^+^ microbeads (MACS, Miltenyi Biotec, #130-117-044) as per the manufacturer's protocol. Isolated populations were routinely >96% CD8^+^. CD8α^+^ enriched cells from the OT-I mice (1 × 10^5^ cells/well) were cultured with high (10^−7^M), low (10^−11^M) or no (NS) OVA_257−264_ pulsed stimulators (1 × 10^6^ cells/well) in the presence of 10 U/ml of rhIL-2 in 96-well flat bottom plates for 48 h at 37°C in a 5% CO_2_ humidified incubator. Supernatants were harvested at 48 h post stimulation and IL-4 production assessed via ELISA.

### Flow Cytometric Detection of IL-4Rα and Phosphorylation of STAT6

OT-I Rag2^−/−^ splenocytes were co-cultured with irradiated C57BL/6 OVA_257−264_ peptide-pulsed splenocytes (1:10 ratio) with 10 U/ml of rhIL-2 for the IL-4Rα assay and in the absence of rhIL-2 for the pSTAT6 assay. CD8^+^ T cells were harvested at 5, 24, and 48 h post primary and secondary stimulation. For surface analysis of IL-4Rα, cells were pelleted and stained with Zombie Aqua fixable viability kit (Biolegend) followed by AF488 anti-mouse CD8α (clone-53-6.7) (Biolegend) and PeCy7 anti-mouse IL-4Rα (clone-I015F8) (Biolegend) or PeCy7 rat IgG2b, k isotype control (clone RTK4530) (Biolegend). For analysis of phosphorylation of pSTAT6, CD8^+^ T cells were fixed with 2% paraformaldehyde (PFA) for 10 min at 37°C to halt phosphorylation events. Cells were washed and then permeabilized with chilled TruePhos perm buffer (Phospho-protein Fixation/Permeabilization Buffer Set) (Biolegend) at −20°C for 60 min. Permeabilized cells were then washed with PBS containing 1% fetal bovine serum and stained with PE rat anti-mouse CD8α (clone 53-6.7)(BD Biosciences) and Alexa Fluor 647 anti-mouse STAT6 (pY641) (BD Phosflow, BD Biosciences) or Alexa Fluor 647 mouse IgG1, k isotype control (BD Phosflow, BD Biosciences). Cells were acquired using the Fortessa X20 (BD Biosciences) and data analyzed using DIVA (BD Biosciences) and FlowJo (BD Biosciences) software.

### Detection of Nuclear NFATC1 and NFATC2 by Fluorescent Microscopy

OT-I Rag2^−/−^ splenocytes (3 × 10^5^ cells) were co-cultured with 5 × 10^6^ irradiated CFSE-labeled C57BL/6 splenocytes that were either peptide-pulsed or left untreated. Cells were cultured with 10 U/ml of rhIL-2 at 37°C in a 5% CO_2_ humidified incubator in 24-well plates containing complete media. OT-I cells cultured in the absence of peptide (non-stimulated) were harvested at 24 h and peptide stimulated OT-I cells were harvested at 48 h post stimulation. Cells (7 × 10^5^) were transferred to poly-L-lysine coverslips and fixed with 2% paraformaldehyde for 20 min. Cells were permeabilized using 0.1% Triton X100 (Fisher Scientific) for 5 min and non-specific binding was blocked using PBS with 10% goat serum (Lampire biological) for 20 min. Cells were stained with purified mouse anti-NFATC1 (Clone-7A6) (BD Pharmingen) for 30 min and then Rhodamine Red-X-conjugated AffiniPure Fab Fragment goat anti-mouse IgG (H^+^L) (Jackson Immuno Research laboratories Inc) for 20 min at room temperature. Cells were then incubated for 20 min with a high concentration (40 μg/ml) of AffiniPure Fab Fragment goat anti-mouse IgG (H^+^L) (Jackson Immuno Research laboratories Inc) to mask mouse IgG epitopes, followed by staining with a second mouse monoclonal antibody to NFATC2 (clone-25A10.D6.D2) (Thermo Fisher Scientific) for 30 min. Antibody was detected with Alexa Fluor 647-conjugated AffiniPure Fab Fragment goat anti-mouse IgG (H^+^L) (Jackson Immuno Research laboratories Inc.). Cells were mounted and nuclei stained with ProLong Gold Antifade reagent with DAPI (Invitrogen). Fluorescent images were collected with a 12-bit Retiga EX 1350 digital camera using a Nikon TE300 microscope equipped with a high-numerical aperture 60X/1.4 oil-immersion objective.

### Metabolic Flux Analysis

OT-I Rag2^−/−^ splenocytes (1 × 10^5^ cells) were co-cultured with irradiated OVA_257−264_ peptide-pulsed splenocytes (1 × 10^6^) in the presence of 10 U/ml of rhIL-2 at 37°C in a 5% CO_2_ humidified incubator_._ Cultures were cultured for 8 h in 96-well plates containing complete media. Cells were then plated in XF culture plates coated with CellTak (Corning) in Seahorse Media (DMEM based media with 10 mM glucose, 2 mM L-Glutamine and 1 mM sodium pyruvate). Oxygen consumption rate (OCR) and extracellular acidification rate (ECAR) were measured under basal conditions and after sequential treatments with 1 μM oligomycin, 1 μM fluoro-carbonyl cyanide phenylhydrazone (FCCP), and 0.5 μM rotenone/antimycin A using a Seahorse XF 96 analyzer (Agilent). Data was analyzed using Wave software according to manufacturer's instructions.

### Metabolic Inhibition Assay

OT-I Rag2^−/−^ splenocytes (1 × 10^5^ cells/well) were co-cultured with irradiated 10^−7^M or 10^−11^M OVA_257−264_ peptide-pulsed C57BL/6 splenocytes or non-peptide pulsed splenocytes (1 × 10^6^ cells/well) in the presence of 10 U/ml of rhIL-2 in 96-well flat bottom plates at 37°C in a 5% CO_2_ humidified incubator. Cells were treated with either 1 μM Oligomycin (Agilent), 10 mM 2-Deoxy-D-Glucose (Sigma Aldrich) or left untreated. Cell supernatants were harvested at 48 h for analysis of IL-4 and TNFα by ELISA. Cell viability was assessed by trypan blue exclusion.

### Statistical Analysis

Statistical analysis was performed using Prism 7 (GraphPad) and the results are represented as the mean ± SEM.

### Quantification of Fluorescent Microscopy

Fluorescent images were processed with the EBImage package (29) in the open source programming environment R (30). Micrographs were acquired with appropriate bandpass filters for DAPI, CFSE, Rhodamine Red-X and Alexa Fluor 647 as 12-bit, 676 × 516-pixel grayscale images. The fluorescent illumination and amplifier settings were adjusted to ensure that the signal for each fluorochrome was acquired in the linear range of the instrument. Raw images were scaled by subtracting the modal value of background pixels in the respective image. Binary nuclear masks defining individual nuclei were generated by applying a local thresholding and watershed algorithm to the low-pass-filtered DAPI image. Nuclear masks along the edge of the image and nuclear masks with an apparent area less than the 5th percentile or greater than the 95th percentile were omitted. Each nuclear mask was enlarged by 15 pixels (3.2 μm) using Voronoi tessellation to create a binary mask defining each cell. The nuclear masks were reduced by 3 pixels (1.2 μm) to define an inner nuclear mask. A perinuclear mask was defined as an annulus of 6 pixels (2.4 μm) surrounding the nuclear border. Registration between the nuclear mask and each fluorescent image was measured and the fluorescent image translated as needed to correct any misalignment. The fluorescent signal within each mask was determined and used for further analysis. CFSE-stained cells were identified and excluded from analysis. Cells out of the plane of focus also were excluded from analysis. These cells were identified as having a perinuclear fraction of DAPI staining that exceeded the median by 1.5-times the interquartile range of this parameter. In three experiments, between 50 and 200 cells could be evaluated for each unique sample. Staining for NFATC1 and NFATC2 was ~2–10-fold greater than staining due to the secondary antibodies alone. The total NFATC1 and NFATC2 signal was normalized to the DAPI signal in each cell and analyzed as log-transformed values. The signal for NFATC1 and NFATC2 in the various masks was measured and expressed as a fraction of the total signal to provide a measure of the cytoplasmic, nuclear, and perinuclear fraction. Because the ratios and log-transformed values were not normally distributed, imaging data were evaluated by non-parametric methods. The Wilcoxon rank sum test was used to detect differences between pairs of samples. Multiple samples were analyzed in two steps. The potential for differences among groups was identified by a significant (*p* < 0.05) outcome with the Kruskal-Wallis omnibus test. Pairwise non-parametric comparisons were then performed by the Dunn test, which adjusts for multiple comparisons (Dunn, 1964). *P* < 0.05 were considered to identify significantly different pairs.

## Results

### Stimulation With a High vs. Low Concentration of Peptide Results in Effector Cells With Disparate Requirements for Peptide That Is Initiated With the First Antigen Encounter and Maintained Over Multiple Stimulations

CD8^+^ effector T cells were generated by weekly restimulation of cells from OT-I Rag2^−/−^ mice with C57BL/6 splenocytes that had been pulsed with a high (10^−7^M) or low (10^−11^M) concentration of OVA_257−264_ peptide. The functional avidity was assessed weekly by quantifying IFNγ production following stimulation with graded concentrations of peptide antigen as we have previously reported (5, 16–20, 31–33). Both of the stimulation conditions resulted in robust recruitment of OT-I cells into the response as demonstrated by expression of CD69 and CD44 as well as recovery and acquisition of effector function (Supplementary Figure 1).

**Figure 1:**
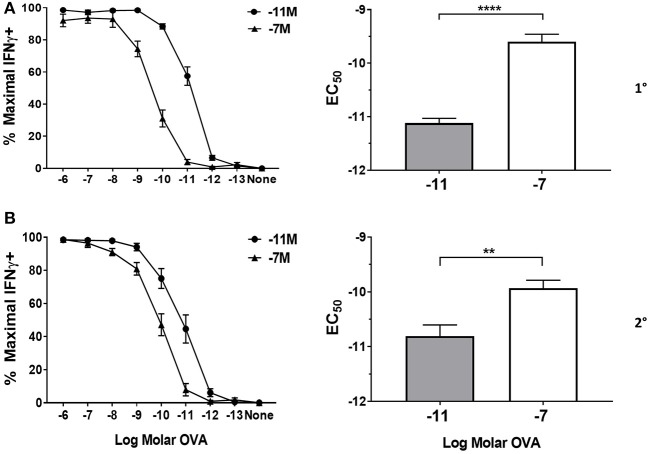
Peptide sensitivity of effector cells is regulated by the amount of antigen used for stimulation. Splenocytes from OT-I Rag2^−/−^ mice were stimulated with irradiated C57BL/6 splenocytes pulsed with a high (10^−7^M) or low (10^−11^M) concentration OVA_257−264_ peptide. As a control, OT-I cells were cultured with splenocytes in the absence of peptide (NS). On day 5 post primary **(A)** and secondary **(B)** stimulation, IFNγ prodcution was assessed by intracellular cytokine staining (ICCS) following stimulation with a range of OVA_257−264_ peptide concentrations (10^−6^M−10^−13^M). The left panel shows the percent maximal IFNγ production (mean ± SEM). The right panel shows the amount of peptide needed to reach half the maximal (EC_50_, mean±SEM) percent of IFNγ-producing cells. Data are from 6 independent cell cultures. Significance was assessed by a two tailed unpaired *t* test. ^**^p < 0.01, ^****^p < 0.0001.

Following primary stimulation, effector cells generated by stimulation with the high peptide condition required significantly more peptide to achieve 50% maximal IFNγ production compared to OT-I cells maintained with low peptide (Figure 1A). The increased requirement of cells maintained on 10^−7^M peptide was apparent throughout the first four stimulation cycles assessed here (Figure 1B and Supplementary Figure 2). Our previous studies suggest that the functional avidity established in these lines becomes a fixed property of the cells that is maintained long-term (our unpublished data). The modulation of functional avidity by peptide in the cultures shown here is in keeping with our previously published lines generated from OT-I or P14 TCR transgenic mice (16–19, 32) as well as WT mice (5).

### High Peptide Stimulation Induces IL-4 Production in OT-I CD8^+^ T Cells

While the ability of high peptide/MHC to program lower avidity is well-established, the mechanism responsible for avidity modulation is unknown. We assessed cytokine production following stimulation with high vs. low peptide/MHC with the hypothesis that encounter with a high vs. low level of peptide/MHC would differentially induce cytokine production by the OT-I cells.

**Figure 2:**
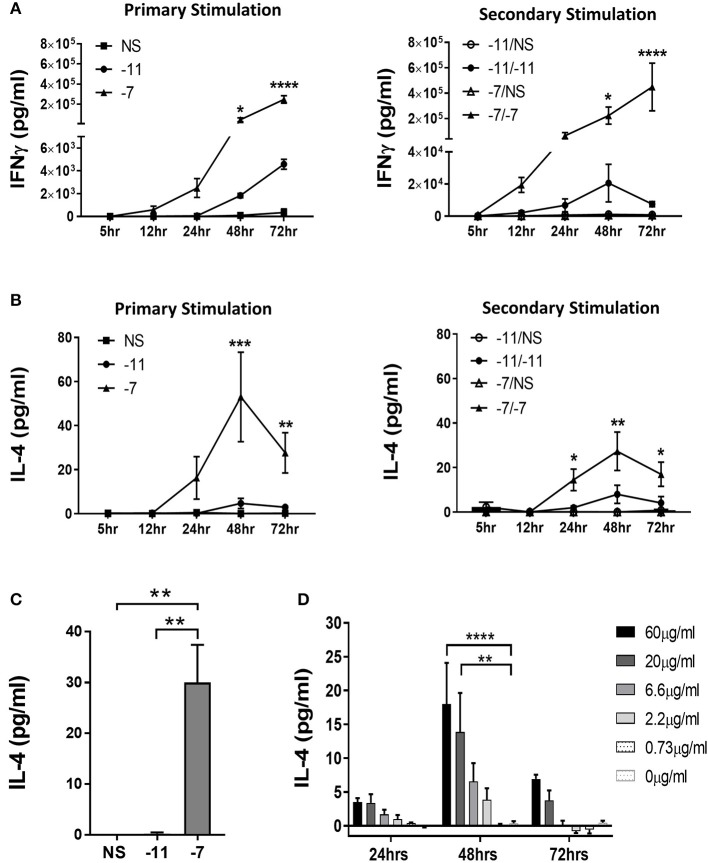
Stimulation with high peptide results in IL-4 production by OT-I cells. Splenocytes from OT-I Rag2^−/−^ mice were stimulated with irradiated C57BL/6 splenocytes pulsed with a high (10^−7^M) or low (10^−11^M) concentration OVA_257−264_ peptide. As a control, OT-I cells were cultured with splenocytes in the absence of peptide (NS). Supernatants were harvested at 5, 12, 24, 48, and 72 h following stimulation. IFNγ **(A)** and IL-4 **(B)** in the supernatant was assessed by ELISA after primary and secondary stimulation. The IFNγ data are the mean±SEM from 3 independent cultures. The IL-4 data are the mean±SEM from 4 independent cultures. Significance was assessed by a two-way ANOVA with multiple comparisons. **(C)** CD8α^+^ OT-I T cells were stimulated with high (10^−7^M), low (10^−11^M) or no (NS) OVA_257−264_ peptide-pulsed stimulators. IL-4 was measured in the supernatant at 48 h by ELISA. The IL-4 data are the mean±SEM from 3 independent cell cultures. Significance was assessed by a one-way ANOVA. ^**^p < 0.01. **(D)** Splenocytes from TCR transgenic OT-I Rag2^−/−^ mice were stimulated with irradiated P815 cells pre-incubated with titrated amounts of anti-CD3ϵ antibody. After 24, 48, and 72 h supernatants were harvested and IL-4 production measured by ELISA. The IL-4 data represent the mean±SEM from 3 independent cultures. Significance was assessed by a two-way ANOVA with comparison to 0 μg/ml. ^*^p < 0.05, ^**^p < 0.01, ^***^p < 0.001, ^****^p < 0.0001.

Splenocytes isolated from naïve OT-I Rag2^−/−^ mice were stimulated with splenocytes previously pulsed with either high (10^−7^M), low (10^−11^M) or no (NS) OVA_257−264_ peptide. IL-4 and IFNγ production were measured at 5, 12, 24, 48, and 72 h post primary stimulation. We also assessed secondary stimulation as an indicator of whether the pattern of cytokine production was maintained. As would be expected, OT-I cells produced IFNγ under both conditions, although the level detected was significantly higher following stimulation with high peptide (Figure 2A). Intracellular cytokine staining (ICCS) analysis at 48 h following stimulation demonstrated that the higher amount of IFNγ in the supernatant was the result of both an increase in the percentage of cells producing IFNγ and the amount produced on a per cell basis (Supplementary Figure 3). IFNγ continued to accumulate in the supernatant through 72 h post primary stimulation in both high and low peptide stimulated OT-I cells. Following secondary stimulation, IFNγ was produced earlier (5–12 h) as would be expected. Similar to the initial stimulation, high peptide resulted in increased IFNγ levels compared to low peptide (Figure 2A).

We next assessed the potential for responding T cells to produce IL-4 in a peptide dose dependent fashion. Significant amounts of IL-4 were detected in cultures stimulated with 10^−7^M peptide-pulsed splenocytes following both primary and secondary stimulation (Figure 2B). IL-4 was initially detected at 24 h and peaked at 48 h. Only minimal IL-4 was present in the cultures maintained on 10^−11^M pulsed stimulators (Figure 2B).

To strengthen our interpretation that the IL-4 was produced by CD8^+^ T cells, we isolated CD8^+^ cells from OT-I splenocytes by magnetic bead selection. Enriched populations were >96% CD8^+^. CD8^+^ OT-I cells were co-cultured with OVA_257−264_ peptide pulsed stimulators. IL-4 was readily detected following stimulation with 10^−7^M, but not 10^−11^M or non-peptide pulsed irradiated splenocytes, supporting the ability of OT-I T cells to produce this cytokine (Figure 2C). These results reveal the production of IL-4 as a previously unknown response of CD8^+^ T cells to stimulation with high amounts of peptide/MHC.

These data suggest that high TCR engagement is the mechanism responsible for the IL-4 production following stimulation with splenocytes pulsed with high peptide. If so, we predicted that exposure to increasing amounts of anti-CD3 antibody would also drive increases in IL-4 production. To test this, naïve OT-I cells were stimulated with titrated amounts of anti-CD3ϵ Ab presented by P815 cells via binding to the FcR, an approach that allows for optimal crosslinking of CD3. IL-4 was measured in the supernatant at 24, 48, or 72 h of culture. We observed a dose dependent increase in IL-4 production, consistent with the high IL-4 produced following stimulation with APC presenting high levels of peptide/MHC (Figure 2D). As with peptide stimulation, IL-4 in the cultures peaked at 48 h. These data establish IL-4 production as a consequence of high TCR engagement on CD8^+^ T cells.

### IL-4Rα Is Not Limiting in CD8^+^ T Cells Stimulated With Low Peptide/MHC

**Figure 3:**
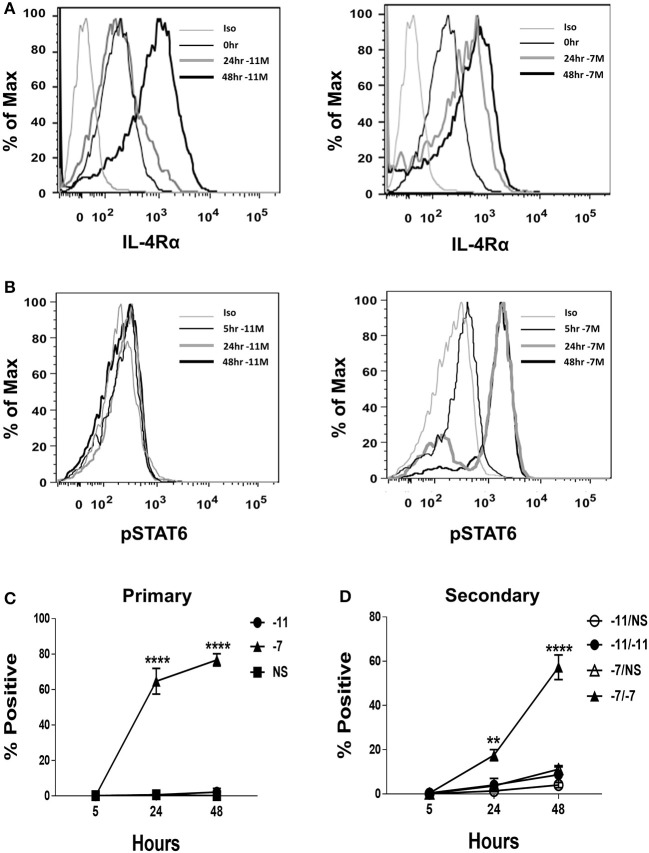
High peptide results in phosphorylation of STAT6. Splenocytes from OT-I Rag2^−/−^ mice were stimulated with irradiated C57BL/6 splenocytes pulsed with a high (10^−7^M) or low (10^−11^M) concentration OVA_257−264_ peptide. As a control, OT-I cells were cultured with splenocytes in the absence of peptide (NS). IL-4Rα was measured at 0, 24, and 48 h following stimulation and stained with anti-IL-4Rα antibody or an isotype control **(A)**. Phosphorylation of STAT6 in OT-I cells was assessed at 5, 24, and 48 h following primary stimulation **(B)**. Representative histogram overlays are shown for the CD8^+^ T cell population. Averaged data (mean ± SEM) for the percentage of cells positive for pSTAT6 following primary **(C)** or secondary **(D)** stimulation from three independent cultures are shown. Significance was assessed by a two-way ANOVA with multiple comparisons. ^**^p < 0.01, ^****^p < 0.0001.

Given the differential production of IL-4 by OT-I cells stimulated with high vs. low peptide/MHC, we considered the possibility that the expression of the IL-4 receptor (IL-4R) may also be regulated by antigen dose in a fashion that impacts the potential for IL-4 to serve in a regulatory capacity. IL-4Rα expression was measured at baseline and following stimulation with high (10^−7^M) or low (10^−11^M) peptide-pulsed stimulators. IL-4Rα was readily detected *ex vivo* in naïve cells (Figure 3A). Stimulation resulted in an increase in IL-4Rα expression regardless of peptide concentration. We did note that while the two stimulation conditions resulted in similar expression of IL-4Rα at 48 h, the kinetics of upregulation differed, with high peptide/MHC stimulation resulting in a more rapid increase in IL-4Rα (Figure 3A). These data suggest the IL-4 pathway is primarily constrained by IL-4 production as opposed to responsiveness that depends on receptor expression.

### CD8^+^ T Cell Encounter With High Peptide/MHC Leads to Increased STAT6 Phosphorylation

Our data thus far show that high peptide increases autocrine IL-4 production and that either high or low peptide activated OT-I cells robustly express IL-4Rα. If the IL-4 produced as a result of high peptide stimulation was contributing to the differentiation of OT-I effectors we would expect to see robust phosphorylation of the IL-4R signaling molecule STAT6. To test this possibility, phosphorylation of STAT6 (pSTAT6) was assessed at 5, 24, and 48 h following stimulation with high (10^−7^M) or low (10^−11^M) peptide-pulsed splenocytes. At 24 h post primary stimulation, on average, nearly 65% of OT-I cells stimulated with high peptide were positive for pSTAT6 and this increased to 77% at 48 h (Figure 3B, right panel and Figure 3C). pSTAT6 was also observed following secondary stimulation (Figure 3D). In contrast, minimal STAT6 phosphorylation was observed in cells stimulated with low peptide (Figure 3B, left panel and Figure 3C). Together, these data show that OT-I cells respond to the IL-4 that they produce following high TCR engagement.

### Autocrine IL-4 Production Drives Low Functional Avidity Following High Peptide/MHC Stimulation

We next tested the potential for high peptide/MHC-induced autocrine IL-4 to modulate functional avidity. While exogenous addition of IL-4 has been reported to decrease avidity in CD8^+^ T cells, the amount utilized in these studies (25 ng/ml) was much greater than that produced by the OT-I cells (34). To determine the modulatory potential of the autocrine IL-4 produced in our cultures, OT-I effectors were generated by weekly stimulation with high (10^−7^M) or low (10^−11^M) OVA_257−264_ pulsed splenocytes in the presence of neutralizing IL-4 antibody. The effectiveness of neutralization was assessed by an ELISA that relied on the same antibody used in the stimulation cultures. We reasoned cytokine binding by the antibody in the culture would prevent IL-4 detection in the ELISA due to competition for the detection antibody in the ELISA. This approach showed an inability to detect IL-4 in the ELISA, consistent with highly efficient blocking of cytokine activity (Supplementary Figure 4A).

**Figure 4:**
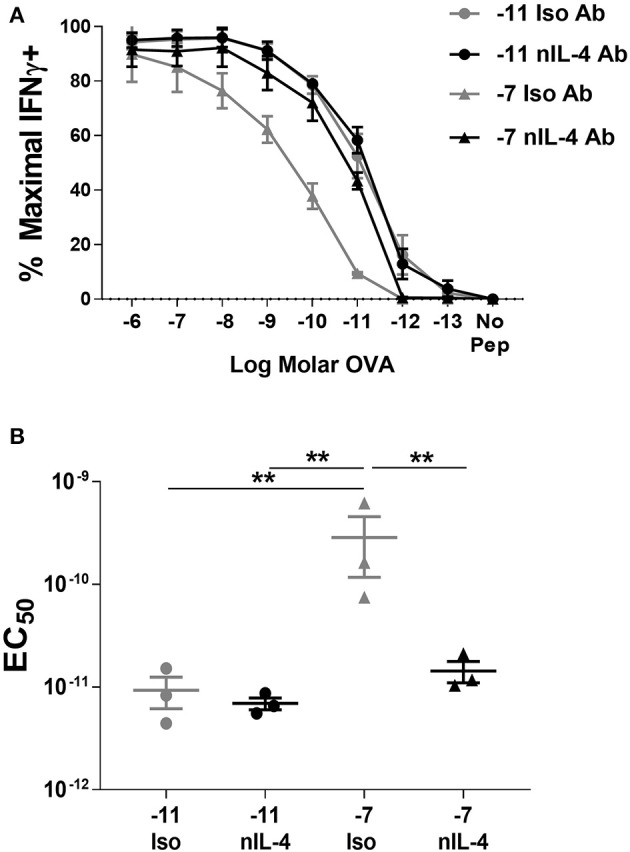
Neutralization of IL-4 in cultures stimulated with high peptide results in an increase in peptide sensitivity. Splenocytes from OT-I Rag2^−/−^ mice were stimulated with C57BL/6 splenocytes pulsed with high (10^−7^M) or low (10^−11^M) OVA_257−264_ peptide. Neutralizing antibody to IL-4 or an isotype control antibody was added (5 μg/ml) at the time of weekly stimulation. On day 5 post quaternary stimulation, function was assessed by stimulating cells with a range of OVA_257−264_ peptide concentrations (10^−6^M−10^−13^M). IFNγ production was assessed from 3 independently generated lines. Data shown are the mean ± SEM percent maximal production of IFNγ **(A)** and the EC_50_ **(B)**. Significance was assessed by a one-way ANOVA with multiple comparisons. ^**^p < 0.01.

Addition of neutralizing IL-4 antibody in the cultures stimulated with high peptide/MHC resulted in a significant increase in peptide sensitivity (Figure 4). In fact, the amount of peptide required to activate the cultures generated by stimulation with high peptide/MHC in the presence of neutralizing IL-4 antibody was similar to that required by effectors generated with 10^−11^M peptide-pulsed splenocytes (Figure 4). In contrast, there was no significant change in functional avidity of cells maintained on 10^−11^M peptide when neutralizing antibody to IL-4 was added to the cultures (Figure 4), as would be expected given the lack of IL-4 production. We also assessed the phosphorylation of STAT6 in cultures that were stimulated with 10^−7^M peptide-pulsed stimulators in the presence of neutralizing IL-4 antibody. In comparison to the isotype control, the average percentage of CD8^+^ T cells positive for pSTAT6 decreased by 4.27 fold in cultures where IL-4 was neutralized (Supplementary Figure 4B). The MFI of the remaining pSTAT6^+^ cells decreased on average by 5.05 fold. Overall these data demonstrate a previously unknown mechanism for self-tuning of the activation setpoint of CD8^+^ T cells that is determined by production of and signaling in response to autocrine IL-4.

### Stimulation With High Peptide/MHC Results in Increased NFAT in the Nucleus

We next sought to understand the effects of high peptide/MHC encounter that resulted in the avidity modulating production of IL-4 in CD8^+^ T cells. In CD4^+^ T cells, IL-4 production is controlled by nuclear factor of activated T cells (NFAT) family members present in the nucleus (35, 36). NFAT proteins are inactive in a phosphorylated form in the cytoplasm; however, when intracellular Ca^2+^ levels rise calcineurin dephosphorylates NFAT and these proteins are translocated to the nucleus where they bind the AP-1 promoter and activate transcription (37). IL-4 transcription in CD4^+^ T cells has been reported to be associated with nuclear NFATC1 (36), while IFNγ appears dependent on NFATC2 (35). To our knowledge, the role of NFAT as a driver of IL-4 production by CD8^+^ T cell has not been previously evaluated.

**Figure 5:**
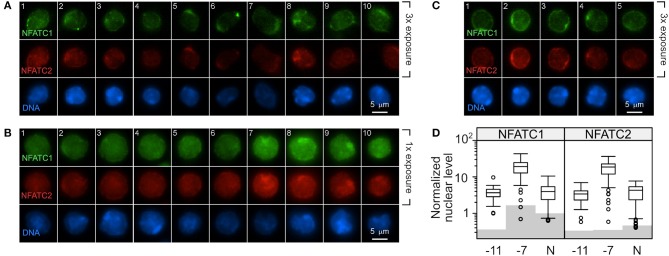
Stimulation with a high concentration of peptide results in increased NFAT in the nucleus compared to stimulation with low peptide. C57BL/6 splenocytes were pulsed with 10^−7^M or 10^−11^M OVA_257−264_ peptide. The irradiated splenocytes were stained with carboxyfluorescein succinimidyl ester (CFSE) and co-cultured with naïve RAG2^−/−^ OT-I splenocytes. After 48 h, cells were stained for DAPI, NFATC1, and NFATC2. In each CFSE-negative cell (OT-I cells), the intensity of the NFATC1 and NFATC2 signal above background was determined and normalized to the DAPI signal in order to permit comparison within each stain. Representative images from two experiments are shown with NFATC1 colored green, NFATC2 colored red and DAPI colored blue. The intensity of the NFATC1 and NFATC2 signal in **(A)** was increased 3-fold over the NFAT signal in **(B)** in order to better visualize the cellular distribution of the respective NFAT protein. OT-I cells stimulated with 10^−11^M peptide-pulsed stimulators are shown in **(A)** and with 10^−7^M in **(B)**. OT-I cells left untreated for 24 h in culture were assessed for nuclear NFAT **(C)**. The average level of nuclear NFATC1 and NFATC2 protein from 50 to 67 cells are shown in **(D)**. Shading indicates the average non-specific signal from the secondary antibody alone for each cell and antibody combination. The non-parametric Dunn's test for multiple comparisons established that the increased nuclear level of NFAT for cells pulsed with 10^−7^M was significantly greater than levels in 10^−11^M cells (5.2–5.5 fold) or non-stimulated cells (4.8–4.3 fold) with an adjusted *p* < 0.001.

OT-I cells were stimulated with high (10^−7^M) or low (10^−11^M) peptide-pulsed splenocytes. OT-I cells cultured in the absence of peptide served as a baseline (Naive, N). The amount of NFATC1 and NFATC2 was evaluated by quantitative fluorescent microscopy 48 h after primary stimulation. Non-stimulated cells were evaluated at 24 h of culture as cell viability was becoming compromised at 48 h. The fluorescent intensity in cells stained with secondary antibody alone served as the background. Cells stimulated with low peptide/MHC (Figure 5A) contained low levels of nuclear NFATC1 and NFATC2. The brightness of the images in Figure 5A was increased 3-fold over those in Figure 5B in order to better visualize the cellular distribution of NFAT. Cells stimulated with 10^−11^M peptide-pulsed splenocytes displayed a perinuclear enrichment of NFAT, which is characteristic of a cytoplasmic protein that is excluded from the nucleus. Further, ~40% of the cells displayed a prominent perinuclear aggregate of NFAT protein as seen in the representative cells in panels 1, 2, 5, and 6 of Figure 5A and panels 2, 3, and 4 of Figure 5C. Stimulation with high peptide/MHC increased the levels of NFATC1 and NFATC2 present at 48 h (Figure 5B). Prominent perinuclear aggregates of NFAT protein were observed in <10% of these cells. In the absence of stimulation, low levels of nuclear NFATC1 and NFATC2 were observed (Figure 5C). The brightness of the images for both non-stimulated and 10^−11^M stimulated OT-I cells was increased 3-fold to facilitate visualization of NFAT. The localization of NFAT protein was determined with an annulus extending from 1.2 μm within the nuclear border to the periphery of the cell. A non-parametric Dunn's test for multiple comparisons established that the increased nuclear levels of NFATC1 and NFATC2 for cells pulsed with 10^−7^M was significantly greater than levels in 10^−11^M cells (5.2–5.5-fold) or naïve cells (4.8–4.3-fold) with an adjusted *p* < 0.001 (Figure 5D).

### Autocrine IL-4 Production Is Dependent on Calcium

**Figure 6:**
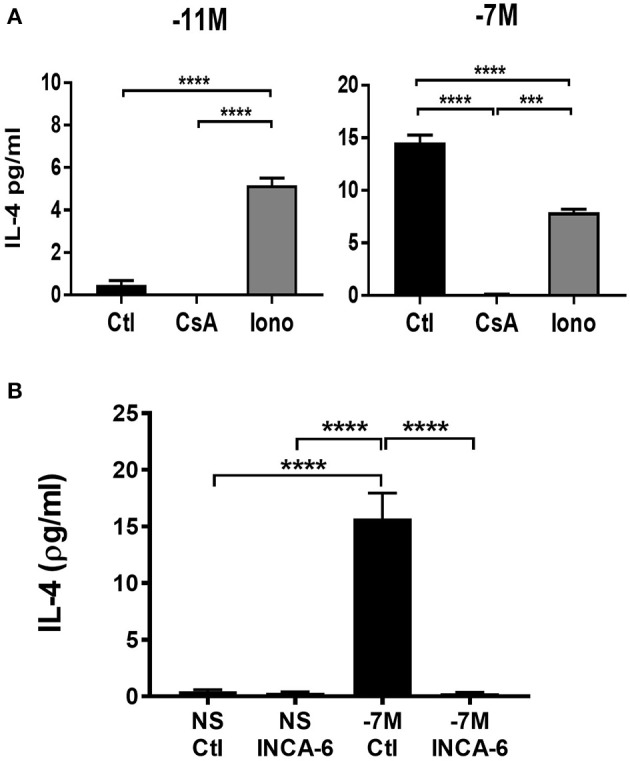
IL-4 production is associated with increased signaling through the calcium pathway. Cyclosporine A (CsA) (100 ng/ml), ionomycin (Iono) (500 ng/ml) or vehicle control-DMSO (Ctl) (0.05%) was added to OT-I Rag2^−/−^ splenocytes during stimulation with splenocytes pulsed with either high (10^−7^M) (**A**, right panel) or low (10^−11^M) (**A**, left panel) concentration of OVA_257−264_ peptide. INCA-6 (25 μM) or vehicle control-DMSO (0.014%) was added to OT-I Rag2^−/−^ splenocytes during stimulation with splenocytes pulsed with high (10^−7^M) peptide or left unstimulated **(B)**. After 48 h supernatants were harvested and IL-4 production measured by ELISA. The data shown is the amount of IL-4 in the supernatant from 3 independent experiments (mean ± SEM). Statistical significance was assessed using a one-way ANOVA with multiple comparisons. ^***^p < 0.001, ^****^p < 0.0001.

The increased nuclear NFATC1 and perhaps NFATC2 in cells stimulated with high peptide/MHC is consistent with a model wherein IL-4 production is a downstream consequence of the increased presence of these transcription factors in the nucleus. We hypothesized that IL-4 production would be altered by modulating signaling through the calcium/calcineurin pathway. Naïve OT-I cells were stimulated with high (10^−7^M) or low (10^−11^M) peptide-pulsed splenocytes in the presence of cyclosporine A (CsA) to inhibit calcineurin or ionomycin to increase intracellular Ca^2+^. The effect on IL-4 was assessed at 48 h following stimulation. Cells that were stimulated with high peptide/MHC in the presence of CsA exhibited a significant decrease in IL-4 production when compared to the vehicle control (Figure 6A, right panel), while cells that were stimulated with low peptide/MHC in the presence of ionomycin had significantly increased production of IL-4 (Figure 6A, left panel).

To confirm that IL-4 production was a downstream consequence of increased NFAT activation we used a small molecule inhibitor know as INCA-6 which selectively binds with high affinity to calcineurin, blocking calcineurin dependent NFAT dephosphorylation (38). Unlike CsA, INCA-6 does not interrupt any other downstream calcineurin signaling [e.g., IκBα kinase (IKIC) and NFKβ (39, 40) other than NFAT (38). Autocrine IL-4 was measured in the supernatants of cells at 48 h following stimulation with or without INCA-6. Cells that were stimulated with high peptide in the presence of INCA-6 exhibited a decrease in IL-4 production compared to high peptide stimulated cells with a vehicle control (Figure 6B). These data confirm the effects of CsA.

### High Peptide Stimulation Results in Metabolic Reprogramming That Controls Autocrine IL-4 Production

Our data show that stimulation with high peptide increases NFAT activation and IL-4 production that is mediated by regulated cytosolic calcium flux. The coordination of these two processes is highly dependent on mitochondrial and glycolytic metabolism. Previous studies show that mitochondria localize to the immunological synapse during T cell activation, where these organelles regulate calcium flux, resulting in NFATC1 activation (41). Further, T cell stimulation is known to enhance nutrient uptake and breakdown of glucose as well as enzymatic activity of key enzymes in the TCA cycle, thereby increasing mitochondrial respiration (42). Based on this understanding, we predicted that stimulation with high peptide/MHC would result in an altered metabolic profile compared to stimulation with low peptide/MHC.

**Figure 7:**
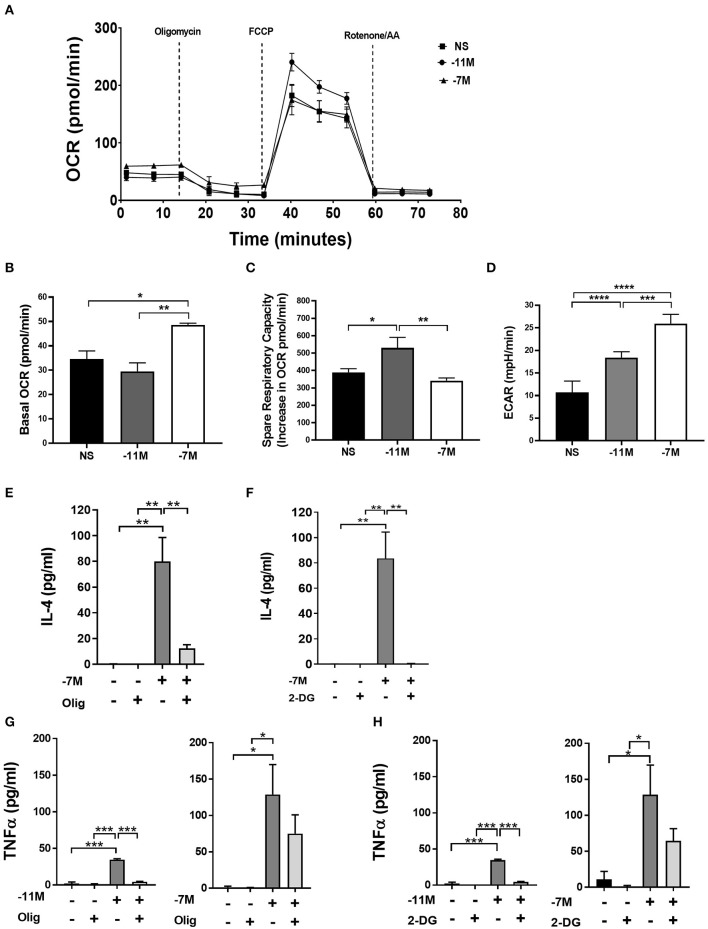
Stimulation with high vs. low peptide results in differences in metabolic reprogramming of cells. Splenocytes from OT-I Rag2^−/−^ mice were stimulated with irradiated C57BL/6 splenocytes pulsed with a high (10^−7^M) or low (10^−11^M) concentration OVA_257−264_ peptide. As a control, OT-I cells were cultured with splenocytes in the absence of peptide (NS). OCR was measured in real time under basal conditions and in response to sequential injection of oligomycin (ATPase inhibitor), FCCP (mitochondrial membrane uncoupler), rotenone/AA (Inhibitor of complex I and III of the ETC). Data in A show the bioenergetics from a representative experiment that included 4 replicates, mean ± SEM bioenergetic profile. Basal mitochondrial function was calculated by subtracting non-mitochondrial respiration (O_2_ consumption after injection of rotenone/AA) from basal O_2_ consumption, *n* = 3 **(B)**. The percent of spare respiratory capacity is calculated as the increase in O_2_ consumption after FCCP injection (Maximal respiration) divided over the basal OCR **(C)**. ECAR was measured as a surrogate of glycolytic flux (due to the increase in lactate) *n* = 3 **(D)**. Averaged data (mean± SEM) from 3 independent cultures (4 replicates per experiment). OT-I cells were stimulated with high (10^−7^M), low (10^−11^M), or no (NS) OVA_257−264_ peptide/MHC. Cultures were treated with or without oligomycin (1 μM) or 2-DG (10 mM); after 48 h IL-4 (Olig, **E**) (2-DG, **F**) and TNFα (Olig, **G**) (2-DG, **H**) was measured in the cell supernatant via ELISA, for NS cultures *n* = 3, for 10^−11^M cultures *n* = 2, for 10^−7^ cultures *n* = 3–5. Statistical significance was assessed by a one-way ANOVA with multiple comparisons. ^*^p < 0.05, ^**^p < 0.01, ^***^p < 0.001, ^****^p < 0.0001.

Oxidative phosphorylation and glycolysis were measured 8 h after stimulation with splenocytes pulsed with high (10^−7^M) or low (10^−11^M) peptide or splenocytes without peptide (NS). High peptide stimulation resulted in a 40% (^**^p < 0.007) increase in basal oxygen consumption rate (OCR) when compared to low peptide and an over 30% (^*^p < 0.03) increase when compared to non-stimulated cells. In stark contrast, cells stimulated with the low peptide condition did not increase oxidative phosphorylation compared to the non-stimulated cells. This suggests that high peptide exposed OT-I cells have selectively elevated levels of oxidative phosphorylation compared to low peptide exposed cells (Figures 7A,B). Interestingly, the spare respiratory capacity was higher in low peptide stimulated OT-I cells when compared to the rest of the groups (Figure 7C). Cells stimulated with the low concentration of peptide also exhibited a significant increase in the percent maximal respiration (normalized to baseline OCR) (Supplementary Figure 5A).

To examine the potential for a shift in glycolysis we evaluated glycolytic flux by measurement of the extracellular acidification rate (ECAR). Our data indicated that high peptide stimulated cells had an over 40% (^**^p < 0.001) increase in ECAR when compared to low peptide stimulated cells and an over 60% (^***^p < 0.0001) increase when compared to non-stimulated cells (Figure 7D). Low peptide stimulation of OT-I cells also increased glycolysis, but this was significantly decreased compared to cells exposed to high peptide. While there was an overall greater increase in metabolic activity in the 10^−7^M stimulated cells compared to 10^−11^M stimulated cells, the OCR/ECAR ratio was not significantly different at this time point, suggesting there was no peptide concentration dependent preferential upregulation of the two metabolic pathways (Supplementary Figure 6).

The altered metabolic profile observed with increased peptide stimulation led us to investigate the effects of OXPHOS and glycolysis on the autocrine production of IL-4. OT-I cells were cultured with irradiated splenocytes previously pulsed with high (10^−7^M) or low (10^−11^M) peptide or no peptide (NS). Cells were treated with either 1 μM of oligomycin, a known inhibitor of OXPHOS through the inhibition of ATP synthase, or 10 mM of 2-Deoxy-D-glucose (2-DG), a glucose analog that inhibits glycolysis. Production of autocrine IL-4 was measured at 48 h. The inhibition of OXPHOS in cells stimulated with high peptide resulted in a near complete loss of IL-4 production when compared to the untreated control (Figure 7E). A similar effect was observed when glycolysis was inhibited (Figure 7F). Treatment with 2-DG or oligomycin did not result in a reduction in the cell counts, showing that the decrease in IL-4 production was not due to decreased cell viability (Supplementary Figure 5B).

We also measured the effect of these treatments on the production of TNFα. Cells stimulated with high peptide in the presence of oligomycin, while trending toward a reduction, showed no significant decrease in cytokine production compared to cells without treatment (Figure 7G, right panel). In contrast, cells stimulated with low peptide had a near complete loss of TNFα production (Figure 7G, left panel). Inhibition of glycolysis also resulted in a near complete inhibition of TNFα production in cells stimulated with low peptide (Figure 7H, left panel), while having no significant effect on those stimulated with high peptide (Figure 7H, right panel). Together, these data show IL-4 production is highly dependent on both OXPHOS and glycolysis as blocking either pathway results in the near complete loss of IL-4 production. While TNFα production following stimulation with the low amount of peptide was also dependent on both the glycolytic and OXPHOS pathways, inhibiting either of these pathways individually in OT-I cells stimulated with high peptide did not result in loss of the ability to produce TNFα. These data suggest the metabolic regulation of cytokine production diverges in CD8^+^ T cells depending on the strength of stimulation.

## Discussion

Here we identify a previously unknown autocrine mechanism through which CD8^+^ T cells sense and respond to the level of antigen by reprogramming metabolism, resulting in IL-4 production. IL-4 acts as an autonomous tuning molecule that establishes the cell's sensitivity to subsequent TCR engagement, i.e., functional avidity. The changes in metabolism induced by high antigen include a significant increase in oxidative phosphorylation in addition to the expected increase in glycolysis in naïve cells undergoing activation (4). Autocrine IL-4 production by CD8^+^ T cells is dependent on both of these metabolic pathways.

Our finding that CD8^+^ T cells can discriminate the peptide/MHC level encountered during activation and respond by producing IL-4 that tunes their antigen sensitivity is a significant step forward in our understanding of the ability of CD8^+^ T cells to respond to environmental signals. The observation that CD8^+^ T cells can produce IL-4 is in agreement with other reports (25, 43–45); however this is, to our knowledge, the first demonstration of an antigen dependent mechanism through which this function is regulated. The ability of IL-4 to act as a regulator of CD8^+^ T cell function has been reported in models of exogenously added IL-4 (22–27, 34, 46–52). Interestingly, in these cases addition of exogenous IL-4 often results in the reduction/loss of IFNγ production concurrent with the acquisition of IL-4 (22, 34, 46, 48). This is in stark contrast to the cells generated in our cultures in the presence of autocrine production of IL-4, as they continue to produce high levels of IFNγ. The loss of IFNγ production in previous studies may be a result of the much higher amounts of IL-4 to which the cells were exposed compared to the level produced by the CD8^+^ T cells in our cultures. Thus, the more physiologic level associated with autocrine production of IL-4 appears to preserve this important effector function.

The question arises as to why CD8^+^ T cells would possess this autocrine regulatory mechanism. IL-4 producing-CD8^+^ T cells elicited *in vivo* have been identified in chronic graft vs. host disease (45). These T cells tend to co-express IFNγ similar to what we observed in our studies. Thus, one potential role for acquisition of the ability to produce IL-4 is dampening of immune responsiveness in situations where tolerance is needed. IL-4 producing CD8^+^ T cells have been observed in other models of chronic immune stimulation including arthritis and lymphoma (43, 44). Further, IL-4 production by CD8^+^ T cells may impact other immune processes. In humans, a population of IL-4 producing CD8^+^ T cells has been reported within germinal centers (53). These cells exhibited Tfh like function in their ability to promote antibody production suggesting IL-4 production by CD8^+^ T cells may have a role in antibody regulation. Finally, there is evidence that IL-4 producing CD8^+^ T cells are generated during acute infectious processes. A subpopulation of cells elicited by fowlpox infection of mice were positive for IL-4 mRNA (25). The data presented here provide a potential mechanism for the acquisition of IL-4 production in CD8^+^ T cells.

IL-4 production following high peptide/MHC encounter by CD8^+^ T cells is correlated with prominently increased levels of the transcription factors NFATC1 and NFATC2. Previous studies have demonstrated the important role of peptide level in regulating calcium signaling in CD8^+^ T cells (54), which is a potent activator of NFAT. NFATC1 and NFATC2 have been primarily studied in the regulation of CD4^+^ T cell subset differentiation. The presence of high levels of NFATC1 has been associated with transcription of TH2 cytokines (36, 55). Further, CD4^+^ T cells deficient in NFATC1 exhibited decreased IL-4 secretion (36). NFATC2 appears to act to oppose IL-4 production in CD4^+^ T cells as inhibition of NFATC2 activity through expression of an inactive NFATC2 resulted in increased IL-4 production (56). In agreement, a study by Kiani et al. reported increased IL-4 production in NFATC2^−/−^ CD4^+^ T cells (57). A follow-up study from this group showed NFATC2 promotes down-regulation of IL-4 gene transcripts *in vitro* and *in vivo* (35). The role of NFATC1 vs. NFATC2 in regulating IL-4 production has not been evaluated in CD8^+^ T cells. Both transcription factors appear to contribute to IFNγ gene transcription (58, 59). Our data strongly support an increase in NFATC1 in the nucleus in cells stimulated with high peptide/MHC. This would be in agreement with the IL-4-promoting role for NFATC1 in TH2 cells. While we observed a trend in NFATC2, we cannot conclude whether it is significantly increased. Defining the potential differential roles of these two factors requires further study.

Our analysis of the metabolic programming that results from encounter with high vs. low peptide/MHC revealed an exciting new mechanism that contributes to the regulation of IL-4 production. Previous data have led to a model wherein the metabolic profile of a naïve T cell shifts from predominantly OXPHOS to glycolysis following activation (4). This metabolic switch results in increased production of ATP that is needed for cells to generate additional energy to support proliferation and effector function (2, 4, 60, 61). Our data show that naïve CD8^+^ T cells triggered by high amounts of peptide/MHC exhibit an increase in both mitochondrial respiration and glycolysis when compared to cells stimulated with low peptide/MHC. The increase in NFATC1 following stimulation with high peptide is in agreement with the finding that NFATC1 is an important determinant of the switch from OXPHOS to glycolysis in CD8^+^ T cells (58). Glycolysis is proposed to act as rheostat for cytokine production, tuning the amount of translation to match the metabolic state of the cell (2, 61). The potential for glycolysis to modulate cytokine production is linked to the post-transcriptional regulation of cytokine message, which occurs through the release from lactate dehydrogenase-mediated repression of AU-rich elements present in cytokine transcripts (61). The dependence of IL-4 production on the glycolytic pathway is consistent with these findings.

Our studies also revealed a dependence on increases in OXPHOS for IL-4 production by the CD8^+^ T cells stimulated with high peptide/MHC. As noted above, the prevailing view is that it is the increased aerobic glycolysis that occurs as CD8^+^ T cells transition to effectors that enables these cells to produce cytokines. Our data expand this paradigm, showing that IL-4 cytokine production by cells undergoing differentiation to effector cells can also be regulated by changes in OXPHOS. The increase in OXPHOS selectively in cells that were activated by encounter with high peptide/MHC acts as a gatekeeper for the production of this immunoregulatory cytokine. Interestingly, the contribution of OXPHOS to cytokine production may be more prevalent than previously thought as we also saw a dependence on the baseline level of this pathway for the production of TNFα in OT-I cells stimulated with the low amount of peptide. With that said, high peptide stimulation impacts the contribution of these pathways to TNFα production as blocking either individually does not prevent production of this cytokine. These findings demonstrate a differential reliance of these pathways dependent on the strength of stimulation.

Interestingly, a role for OXPHOS has been reported during the differentiation of naïve CD4^+^ T cells along the regulatory pathway (62, 63). Inducible Tregs (iTreg) generated by stimulation of naïve CD4^+^ T cells in the presence of TGFβ exhibited elevated lipid oxidation compared to cells cultured with Th1, Th2, Th17 polarizing conditions (62). iTreg development was dependent on this increase in OXPHOS as addition of the inhibitor of the carnitine palmitoyl-transferase-1 inhibitor Etomoxir (Etx), which blocks mitochondrial lipid uptake and oxidation, impeded iTreg generation. Treatment with Etx did not impact differentiation of other T effector cell populations (62). This dependence on OXPHOS for iTreg differentiation is not observed for thymic Tregs which rely on glycolysis (64). Together with our data, these findings support the use of the OXPHOS pathway in T cells as a mechanism to drive differentiation of T cells along pathways that dampen the immune response, either through Treg differentiation or decreased sensitivity.

The ability of T cells to tune for optimal responsiveness to dangerous pathogens while simultaneously dampening unwanted responses is critical to the survival of the host. This view is supported by a significant body of work showing CD8^+^ T cell sensitivity to peptide/MHC is a critical determinant of *in vivo* efficacy in models of tumor immunity and pathogen clearance (5–12). The data presented here reveal a new paradigm for regulating this important property. We find that peptide/MHC sensitivity can be controlled by a self-tuning event through the action of autocrine IL-4, the production of which is dependent on a metabolic state that includes high OXPHOS. This required metabolic reprogramming in activated cells is regulated by TCR signaling strength. These studies are significant as they expand our model for metabolism driven regulation of CD8^+^ effector cell differentiation and function. While important for our understanding of optimal responsiveness to antigen, our results also have implications for peripheral tolerance, given the established induction of tolerance that can occur in CD8^+^ T cells as a result of encounter with high peptide (65). In summary, these results identify a new mechanism for autonomous CD8^+^ T cell tuning of their activation threshold that is dependent on IL-4. Further, they show that the production of this important modulatory signal is controlled by metabolic reprogramming in the responding effector cell.

## Data Availability Statement

The datasets generated for this study are available on request to the corresponding author.

## Ethics Statement

The animal study was reviewed and approved by Wake Forest Animal Care and Usage Committee, Wake Forest School of Medicine.

## Author Contributions

KC and MA-M designed experiments, analyzed data and wrote the manuscript. DO designed and analyzed data from the microscopy study. KC and BH performed experiments. DS-P performed the Seahorse analysis and analyzed the data. DO and DS-P evaluated and edited the manuscript.

### Conflict of Interest

The authors declare that the research was conducted in the absence of any commercial or financial relationships that could be construed as a potential conflict of interest.
