# Supplementary material for: TCR Dependent Metabolic Programming Regulates Autocrine IL-4 Production Resulting in Self-Tuning of the CD8+ T Cell Activation Setpoint
Source: Front Immunol. 2020 Mar 31;11:540. doi: 10.3389/fimmu.2020.00540 (PMC7145404; doi:10.3389/fimmu.2020.00540)
Supplement: Supplementary file 1 [file Presentation_1.pdf]

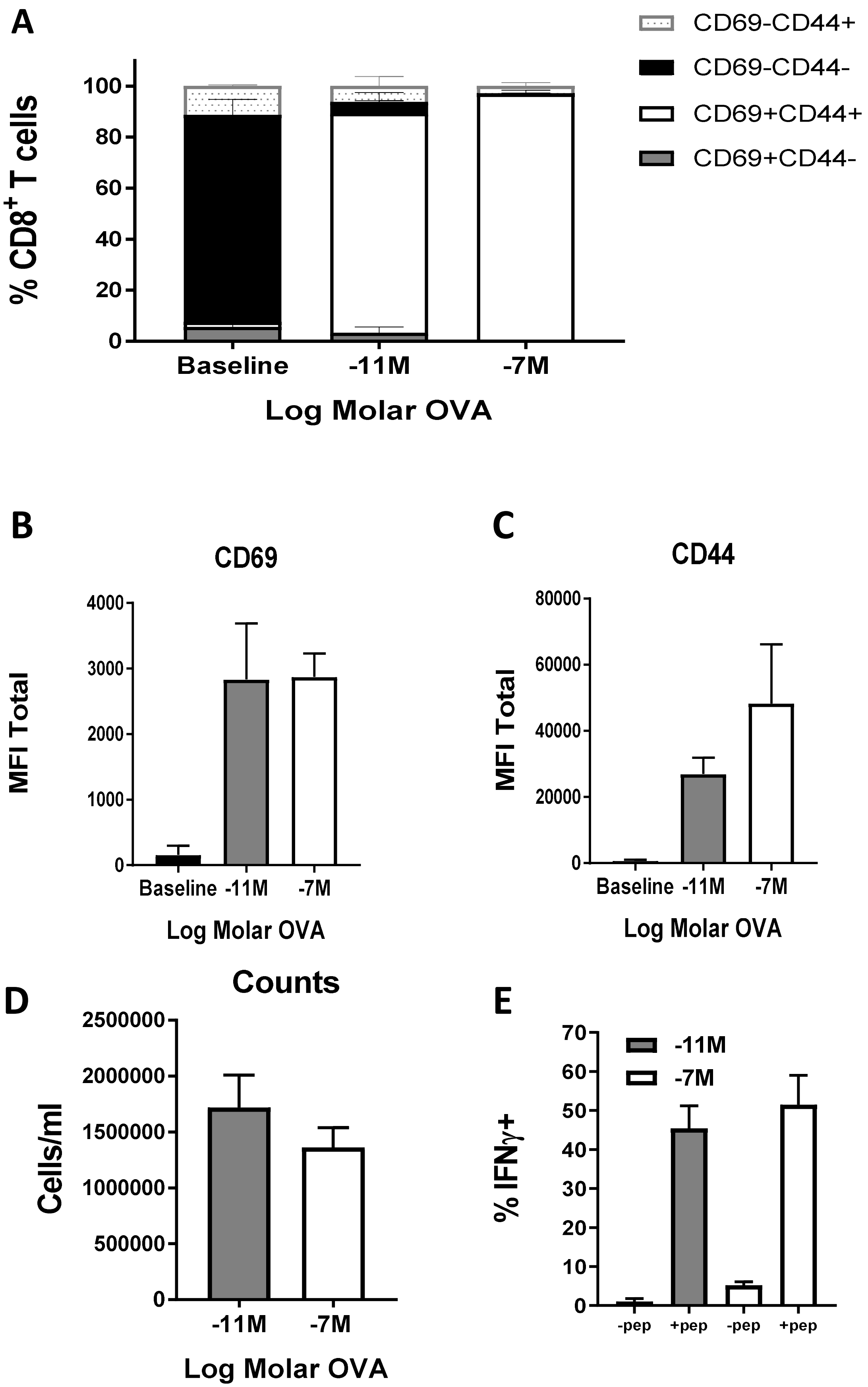
A
CD69-CD44+
CD69-CD44-
CD69+CD44+
CD69+CD44-
% CD8+ T cells
100
80
60
40
20
0
Baseline
-11M
-7M
Log Molar OVA
B
CD69
MFI Total
4000
3000
2000
1000
0
Baseline
-11M
-7M
Log Molar OVA
C
CD44
MFI Total
80000
60000
40000
20000
0
Baseline
-11M
-7M
Log Molar OVA
D
Counts
Cells/ml
2500000
2000000
1500000
1000000
500000
0
-11M
-7M
Log Molar OVA
E
% IFNγ+
70
60
50
40
30
20
10
0
-11M
-7M
-pep
+pep
-pep
+pep

Supplementary Fig. 1: **High and low peptide/MHC engagement triggers cell activation after 48hrs of stimulation.** Splenocytes from OT-I Rag2$^{-/-}$ mice were stimulated with irradiated C57BL/6 splenocytes pulsed with high (10^{-7}M) or low (10^{-11}M) $OVA_{257-264}$ peptide. At 48hrs post primary stimulation cell surface CD69 and CD44 expression was measured on OT-I CD8$^+$ T cells by flow cytometry (A). Mean±SEM fluorescent intensity of CD69 (B) and CD44 (C) was assessed. (D) Cell viability was measured 7 days post primary stimulation by counting the number of live cells per ml via trypan blue exclusion, n=6. Data represent mean±SEM . (E) 7 days post primary stimulation, OT-I cultures were restimulated with 10^{-7}M pulsed irradiated stimulators for 5 hrs. Intracellular IFNγ producing OT-I CD8$^+$ T cells were assessed via flow cytometry. All data represent averages from 3 independent cultures.

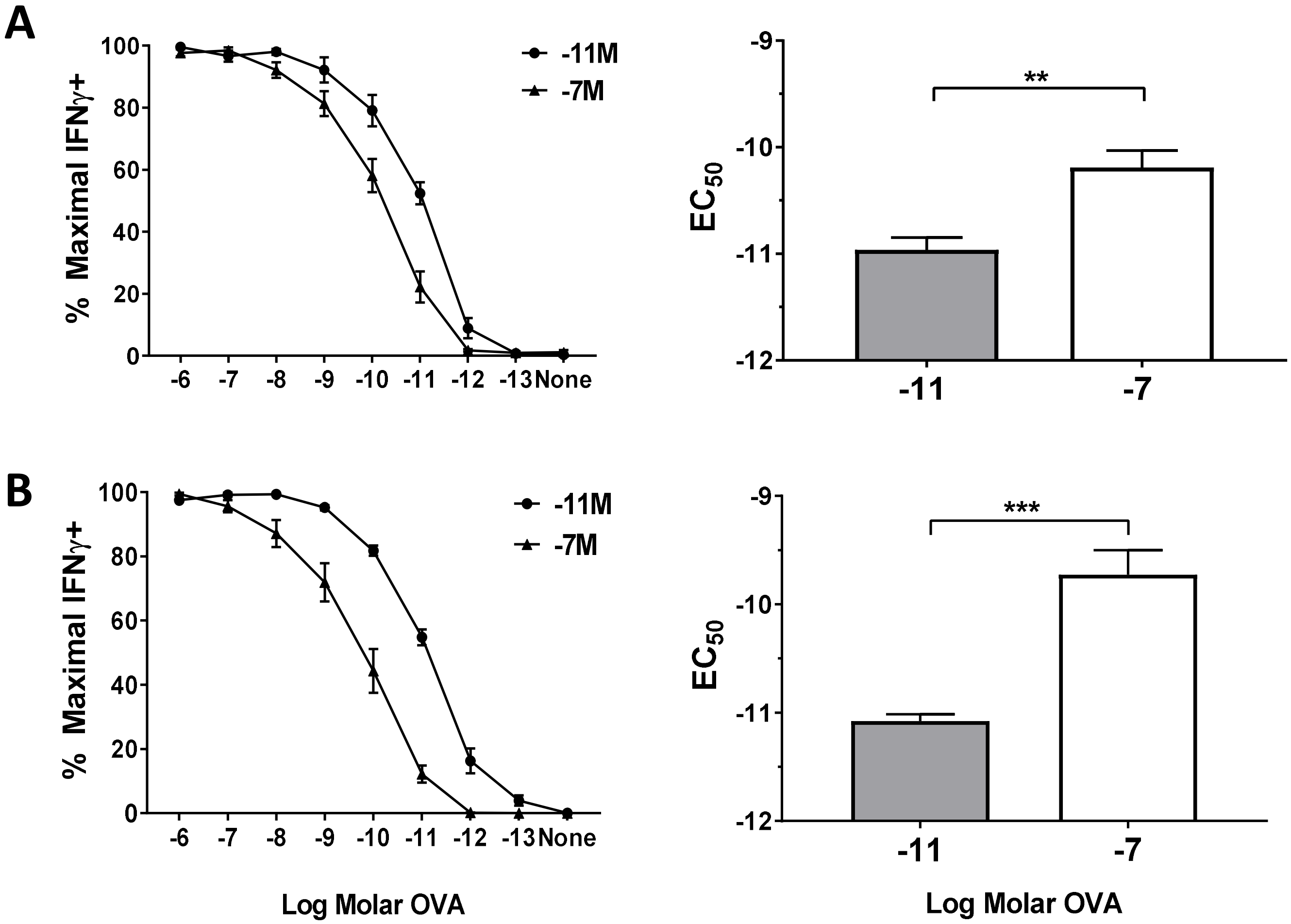

Supplementary Fig 2: Peptide sensitivity of effector cells is regulated by the amount of antigen used for stimulation and is maintained over multiple stimulations. Splenocytes from OT-I Rag2$^{-/-}$ were stimulated weekly with irradiated C57BL/6 splenocytes pulsed with a high (10^{-7}M) or low (10^{-11}M) concentration OVA$_{257\text{-}264}$ peptide. On day 5 post tertiary (A) and quaternary (B) stimulation, function was assessed by ICCS following stimulation with a range of peptide concentrations (n=6). The left panel shows the percent maximal IFNγ production (mean±SEM). The right panel shows the amount of peptide needed to reach half the maximal ($EC_{50,}$ mean±SEM) percent of IFNγ-producing cells. Significance was assessed by a two-tailed unpaired t test. **p = < 0.01,***p = < 0.001.

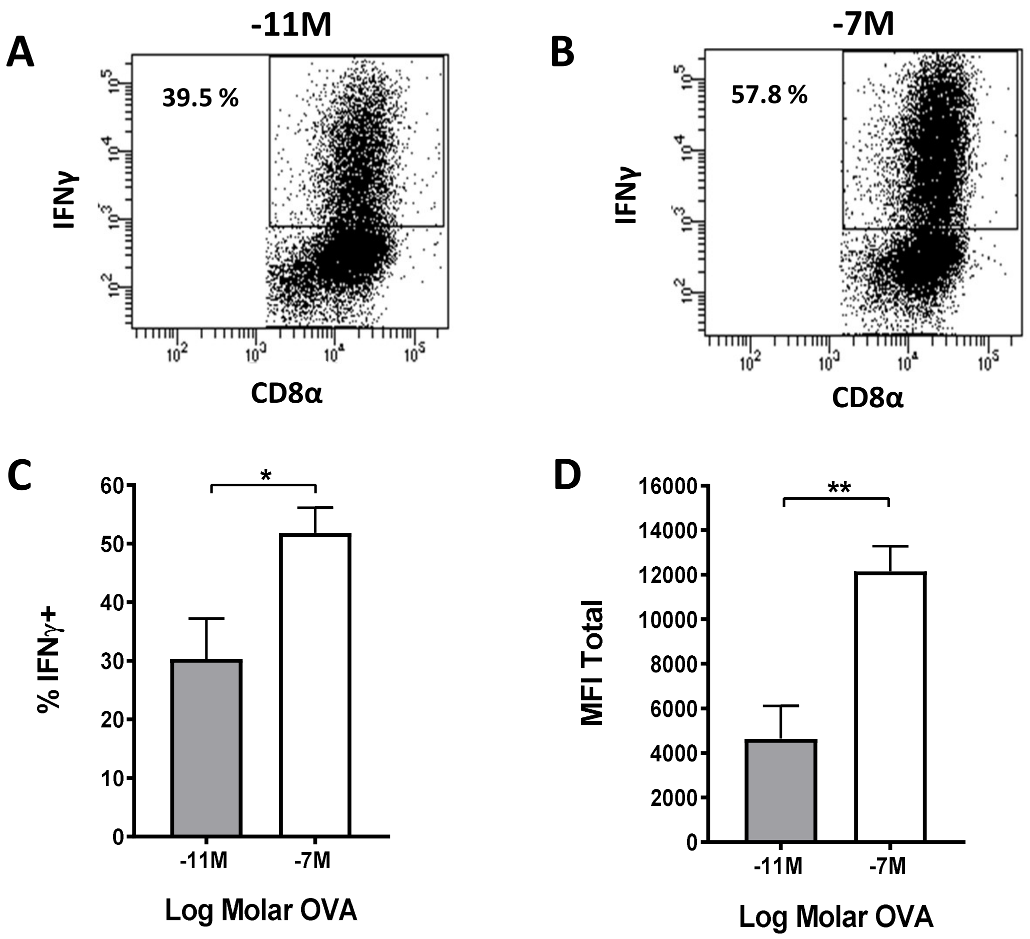

Supplementary Figure 3: **The increased IFNγ detected in culture supernatants following stimulation with high peptide is the combined result of a higher percentage of cells producing IFNγ and a higher amount produced on a per cell basis.** Splenocytes from OT-I Rag2$^{-/-}$ mice were stimulated with irradiated C57BL/6 splenocytes pulsed with high (10^{-7}M) or low (10^{-11}M) $OVA_{257-264}$ peptide. At 48hrs post primary stimulation, brefeldin A was added to the cultures to measure production of IFNγ resulting from the initial activation of the cells. IFNγ was assessed 5 hours later by flow cytometry. Representative plots showing the percent of IFNγ$^+$ CD8α$^+$ OT-I cells pulsed with either low (10^{-11}M) (A) or high (10^{-7}M) (B) $OVA_{257-264}$ peptide. The mean±SEM percent of IFNγ$^+$CD8α$^+$ OT-I cells (C) and IFNγ MFI (D) was determined from 3 independent cultures. Significance was assessed by a one-tailed unpaired t test. * p = < 0.05, **p = < 0.01.

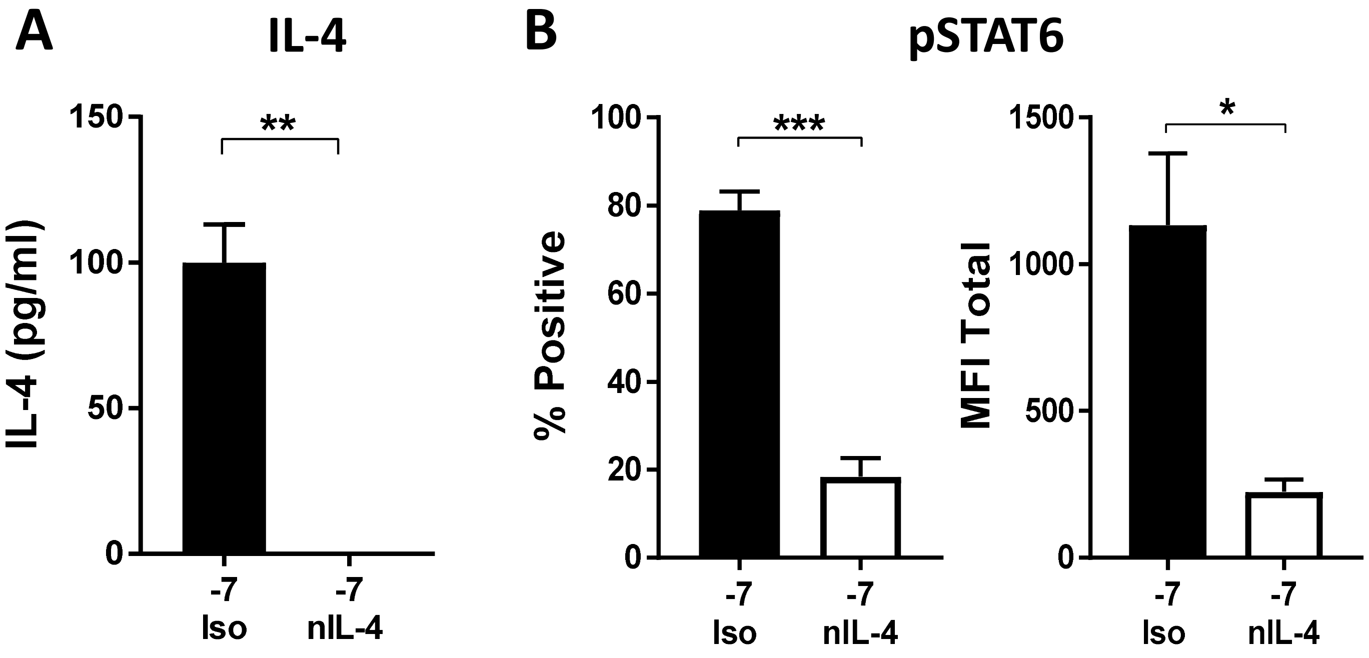

Supplementary Figure 4: Neutralization of autocrine IL-4 results in decreased phosphorylation of STAT6 signaling. Naïve splenocytes from TCR transgenic OT-I Rag2$^{-/-}$ mice were stimulated with irradiated splenocytes pulsed with high (10^{-7}M) $OVA_{257\text{-}264}$ peptide. Neutralizing antibody to IL-4 was added (5 µg/ml) at the initiation of the culture. Supernatants were harvested at 48 hours following primary stimulation. IL-4 production was assessed by ELISA (A). Cells were stained for pSTAT6 and assessed using flow cytometry. Averaged data for the percentage of $CD8^{+}$ T cells positive for pSTAT6 (B left panel) and the MFI of pSTAT6 in the $CD8^{+}$ T cell population (B right panel) was assessed. Data represent the mean±SEM from 3 independent cultures. Statistical significance was assessed using a two-tailed unpaired t test. * p = < 0.05,**p = < 0.01p,***p = < 0.001.

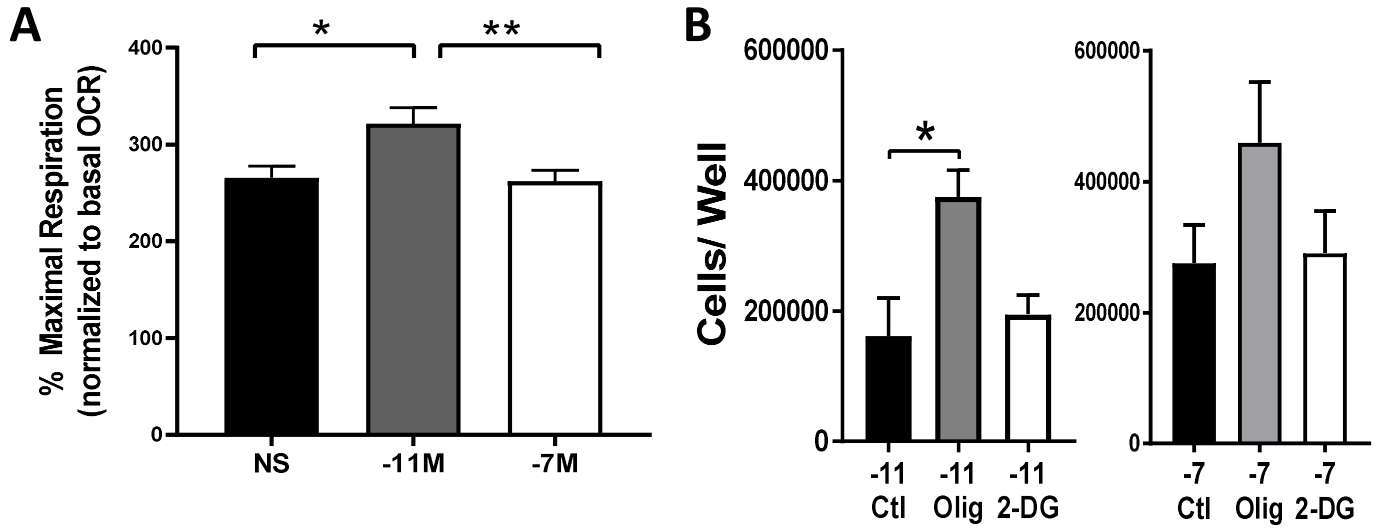

Supplementary Figure 5: Splenocytes from OT-I Rag2$^{-/-}$ mice were stimulated with irradiated C57BL/6 splenocytes pulsed with either a high (10^{-7}M) or low (10^{-11}M) concentration of $OVA_{257-264}$ peptide or left untreated. OCR was measured by seahorse analysis at 8hrs. The data shown are the percent maximal respiration normalized to the baseline OCR level from 3 independent experiments (mean±SEM). The maximal respiration was assessed after normalization to the basal OCR levels. OT-I cells were stimulated with high (10^{-7}M), low (10^{-11}M) or no (NS) $OVA_{257-264}$ peptide/MHC. Cultures were treated with or without oligomycin (1μM) or 2-DG (10mM), cell viability was assessed using Trypan blue exclusion , n=3-5 (B). Statistical significance was assessed by a one-way ANOVA with multiple comparisons. *p = < 0.05, **p = < 0.01. n=3.

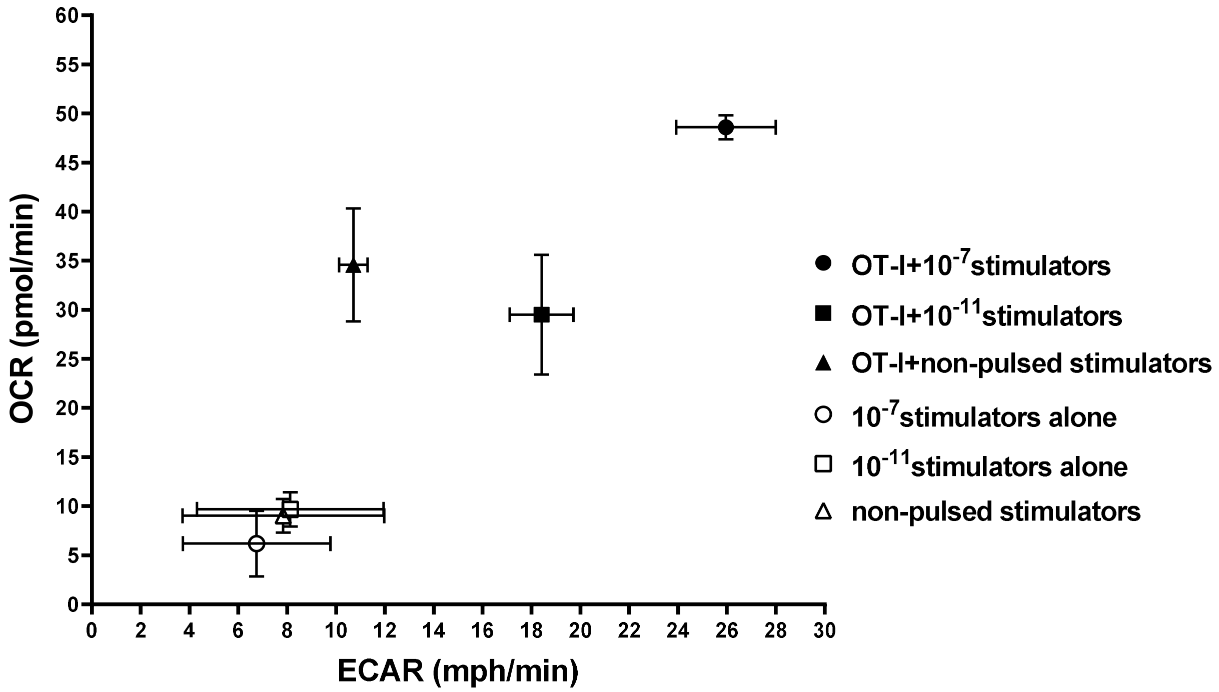

Supplementary Figure 6: The ECAR/OCR ratio is similar in OT-I cells stimulated with high versus low peptide. Splenocytes from OT-I Rag2$^{-/-}$ mice were stimulated with irradiated C57BL/6 splenocytes pulsed with high (10^{-7}M) or low (10^{-11}M) concentrations $OVA_{257\text{-}264}$ peptide or unstimulated. Naïve irradiated splenocytes only were included as a negative control. OCR and ECAR was measured by seahorse analysis at 8hrs, n=3. The irradiated stimulator cells showed low bioenergetic activity and no difference in metabolic rate, indicating that these do not contribute to the bioenergetic profiles of activated T cells**.**
